# Evaluation of the Efficiency of Photoelectrochemical Activity Enhancement for the Nanostructured LaFeO_3_ Photocathode by Surface Passivation and Co-Catalyst Deposition

**DOI:** 10.3390/nano12234327

**Published:** 2022-12-05

**Authors:** Victoria P. Chertkova, Aleksandra N. Iskortseva, Egor M. Pazhetnov, Natalia A. Arkharova, Sergey V. Ryazantsev, Eduard E. Levin, Victoria A. Nikitina

**Affiliations:** 1 Department of Chemistry, Lomonosov Moscow State University, Moscow 119991, Russia; 2 Center for Energy Science and Technology, Skolkovo Institute of Science and Technology, Moscow 121205, Russia; 3 FSRC “Crystallography and Photonics” RAS, Moscow 119333, Russia

**Keywords:** photoelectrocatalysis, photoelectrochemical water splitting, perovskite structure, hydrogen evolution reaction, recombination, charge transfer, co-catalyst

## Abstract

Perovskite-type lanthanum iron oxide, LaFeO_3_, is a promising photocathode material that can achieve water splitting under visible light. However, the performance of this photoelectrode material is limited by significant electron-hole recombination. In this work, we explore different strategies to optimize the activity of a nanostructured porous LaFeO_3_ film, which demonstrates enhanced photoelectrocatalytic activity due to the reduced diffusion length of the charge carriers. We found that surface passivation is not an efficient approach for enhancing the photoelectrochemical performance of LaFeO_3_, as it is sufficiently stable under photoelectrocatalytic conditions. Instead, the deposition of a Pt co-catalyst was shown to be essential for maximizing the photoelectrochemical activity both in hydrogen evolution and oxygen reduction reactions. Illumination-induced band edge unpinning was found to be a major challenge for the further development of LaFeO_3_ photocathodes for water-splitting applications.

## 1. Introduction

Visible light-absorbing transition metal oxides (TMO) have attracted attention as potential active materials for photoelectrochemical (PEC) water splitting and low-carbon fuel production [1,2]. Yet, the practical application of TMO materials in photoelectrocatalysis faces numerous challenges, such as low carrier mobility, high defect densities, short charge carrier lifetimes, charge trapping in polaronic states and modest electrocatalytic activity in multistep reactions of water electrolysis, carbon dioxide reduction or pollutant degradation [3,4,5]. On the other hand, oxide-based materials show promising stability in aqueous solutions, primarily those that are alkaline and neutral. This advantage favorably distinguishes them from III-V semiconductor photoelectrodes, which show high photoelectrocatalytic activity but are hardly stable under reaction conditions [6]. Among TMO materials, perovskites are particularly interesting due to the flexibility of their electronic and crystal structure and their chemical versatility [7,8]. Most of the TMO-based materials, which were extensively studied in photoelectrochemical processes, are photoanodes [1,9,10,11,12,13,14,15,16] (TiO_2_, Fe_2_O_3_, BiVO_4_, SrTiO_3_), while much less attention has been paid to metal oxide photocathodes [17,18,19], except for copper-based materials. Copper-based photocathodes [20], such as Cu_2_O and CuBi_2_O_4_, show attractive activity in photoelectrochemical processes, yet their chemical and electrochemical stability under operating conditions remains a significant challenge [21] since successful application requires surface modification with protective coatings [22].

Fe-based materials (LaFeO_3_ [23,24,25,26], BiFeO_3_ [27], and café_2_O_4_ [28]) were reported to possess rather high stability, which ensures practical interest in this class of photoelectrode materials. However, these materials show quite low external quantum efficiency (EQE) for hydrogen evolution reaction (HER), and that motivates further research aimed at enhancing their PEC performance. *p*-type LaFeO_3_ (LFO) is an attractive perovskite-structured photocathode material, which was reported to be sufficiently stable under the photoelectrolysis conditions in alkaline media, yet shows very modest EQE [17,23,24,25,26,29,30,31,32,33]. Various methods of improving EQE have been proposed, such as doping to tune the electronic structure and affect the charge carrier mobilities [31,33,34], nanostructuring to reduce the diffusion length [23,25], and adding a buffer gold layer to improve charge collection efficiency and charge separation [32]. Still, no consensus currently exists on the optimal strategies of LFO surface modification to enhance the photoelectrochemical activity toward the electrolysis of water. 

Moderately high photocurrents (several hundreds of µA cm^−2^) on LFO films were observed only in experiments with electron scavengers [23,30,34], while the currents for hydrogen evolution are typically much lower. These experimental results imply that the main problem might be rooted in the necessary extension of the lifetime of short-lived photogenerated charges to drive the sluggish water reduction reaction. In electrocatalysis, this problem is solved by selecting highly active electrocatalytic materials, which interact with adsorbates optimally and decrease the activation energy of the limiting step in a multistep reaction. Co-catalyst deposition is a standard approach to enhance the performance of photoelectrodes [35,36], yet most of the data were reported for *n*-type semiconducting photoanodes [37]. Recent studies of the co-catalyst-decorated photoanodes revealed that the role of the co-catalyst in enhancing the PEC performance of a photoelectrode may differ from the expected increase in the charge transfer rate across the photoelectrode/solution interface [37]. For instance, the deposition of a CoPO_4_ co-catalyst on the surface of hematite (α-Fe_2_O_3_) did not lead to the enhancement of charge transfer kinetics, while the improvement in activity was mainly due to the reduced recombination losses [38,39]. A similar study confirmed that catalytic properties of the deposited layer might not be important for the PEC activity of photoanodes, as surface passivation and hence reduced recombination are the predominant sources of PEC activity improvement [38,39,40]. It has also been suggested that a co-catalyst can play both a catalytic role and a non-catalytic one, enhancing not only charge transfer but also the overall stability [40,41], while the predominant effect should be dependent on the nature of the photoelectrode material, the nature of the co-catalyst, as well as on the thickness and uniformity of the co-catalyst layer. It is always problematic to deduce the actual role of the co-catalyst, as one needs to address the competition between charge transfer and electron-hole recombination and understand which of these two factors is altered to a greater extent by the co-catalyst deposition [40]. 

There are far fewer detailed studies [42,43,44] on the co-catalyst effect for the metal oxide photocathodes, which also require co-catalysts to be efficient in multistep proton-coupled electron transfer reactions. Yet, it is of primary importance to formulate optimal strategies for the PEC performance enhancement of promising *p*-type transition metal oxide materials. In this study, we aim to compare the effects of surface passivation and co-catalyst deposition for a nanostructured LFO photocathode to deduce efficient strategies to control and enhance HER kinetics under photoelectrochemical conditions.

## 2. Materials and Methods

### 2.1. Synthesis

#### 2.1.1. LaFeO_3_ Films

Films of LFO on fluorine-doped tin oxide (FTO) substrates were fabricated using a modified procedure from ref. [21]. Briefly, 0.2 g of La(NO_3_)_3_*6H_2_O, 0.19 g Fe(NO_3_)_3_*9H_2_O and 0.38 g of citric acid monohydrate were dissolved in 0.5 mL of deionized water under magnetic stirring at room temperature. After the complete dissolution of the salts, 1 mL of Triton X-100 polymer and 1 mL of acetylacetone was added. The resulting mixture was stirred overnight. The solution was spin coated at 3000 rpm for 30 s onto FTO substrates, which were cleaned in acetylacetone to improve wetting with the sol components. The film was annealed at 500 °C for 20 min to remove the organic components, and the spin-coating procedure was repeated 3 times to obtain a film with an optimal thickness (ca. 300 nm). Finally, the film was annealed at 600 °C for 2 h to obtain the well-crystallized perovskite phase.

#### 2.1.2. Pt Nanoparticles

Citrate-capped Pt nanoparticles were synthesized via a conventional borohydrate reduction route [45]. A total of 1 mL of 16 mM solution of H_2_PtCl_6_ and 1 mL of 40 mM citric acid solution were mixed with 38 mL of deionized water and stirred for 30 min at room temperature. Then, 0.2 mL of a 50 mM NaBH_4_ solution was added dropwise to the mixture, and the color of the solution changed to brownish yellow. The mixture was stirred at ambient temperature for 1 h. The resulting solution was spin coated onto FTO and LFO films for 30 s at 1000 rpm.

#### 2.1.3. TiO_2_ Layers

TiO_2_ layers were deposited onto LFO films via spin coating. Titanium (IV) isopropoxide was mixed with acetylacetone in molar ratios of 1:8 and 1:100 and stirred overnight at room temperature. The resulting sol was spin coated on FTO or FTO/LFO films at 3000 rpm for 30 s. The films were air dried and then annealed at 250 °C to decompose the residual titanium (IV) isopropoxide.

### 2.2. Characterization

X-ray diffraction (XRD) patterns of the films were collected using a Malvern Panalytical Aeris diffractometer (Bragg–Brentano geometry, CuKα radiation, PIXcel^3D^ detector). 

Scanning electron microscopy (SEM) images were registered using an FEI Scios dual-beam scanning electron microscope (field emission gun, landing energy 2 kV, in-lens secondary electrons detector). The specimen cross-section was carried out using a focused ion beam (Ga^+^, 30 kV). To prevent unwanted specimen etching, a Pt protection layer was deposited before cross-sectioning. Transmission electron images were collected in bright-field mode using an FEI Tecnai Osiris transmission electron microscope operated at 200 kV.

Attenuated total reflectance Fourier-transform infrared (ATR-FTIR) spectra (resolution 4 cm^−1^, signal averaging by 20 scans) of the films were measured with a Bruker Alpha II spectrometer equipped with a diamond ATR crystal and a KBr beamsplitter.

UV/Vis/NIR absorption spectra were measured in transmission geometry using a custom setup built with Avantes instruments: an AvaLight-DHc light source (both deuterium and halogen lamps), an AvaSpec-HS2048 spectrometer and fiber-optic light guides. The spectrum of the FTO glass substrate was used as a reference.

X-ray photoelectron spectroscopy (XPS) experiments were performed using a PHI 500 VersaProbe II spectrometer with a spherical mirror analyzer. An Al Kα monochromatic x-ray source with 1486.6 eV X-ray energy was utilized. Survey and high-resolution spectra were recorded with 1.0 eV and 0.1 eV step sizes, respectively. High-resolution XPS spectra were processed to obtain atomic concentrations following a typical procedure for XPS spectra quantifications. Photoelectron backgrounds were subtracted from the high-resolution spectra using Shirley function approximation. The binding energy (BE) of all the spectra were calibrated using the C1s peak from adventitious carbon fixed at 284.8 eV.

### 2.3. Photoelectrochemical Measurements

The photoelectrochemical properties of pristine LFO, LFO/Pt, and LFO/TiO_2_ electrodes were characterized by means of voltammetric and chronoamperometric measurements. The FTO substrates were masked with a non-transparent epoxy resin to expose the area of ca. 0.5–1 cm^2^. All the measurements were performed in a 0.1 M NaOH solution in a PTFE cell with a quartz window and separated graphite counter electrode and FTO/LaFeO_3_ working electrode compartments. HgO/Hg (1 M NaOH) was used as a reference electrode. For the chopped voltammetry and chronoamperometry measurements, a high-power blue LED (ARPL-STAR-3W, λ_max_ = 460 nm) was used as a light source. The sample was illuminated from the front at a light intensity of 45 mW/cm^2^ at 460 nm (as measured by a calibrated silicone photodiode (S121C, Thorlabs) connected to a power meter (PM100A, Thorlabs)). Potentials are reported with respect to the reversible hydrogen electrode (RHE). Prior to the measurements in 0.1 M NaOH, the solution was deaerated with argon for a minimum of 40 min. For the measurements in O_2_-saturated 0.1 M NaOH solution, the solution was purged with O_2_ for 40 min. Electrochemical impedance spectra were recorded under illumination in the frequency range of 100 kHz–10 mHz with a 5 mV alternating potential amplitude.

## 3. Results

### 3.1. Characterization of the LFO Films

**Figure 1:**
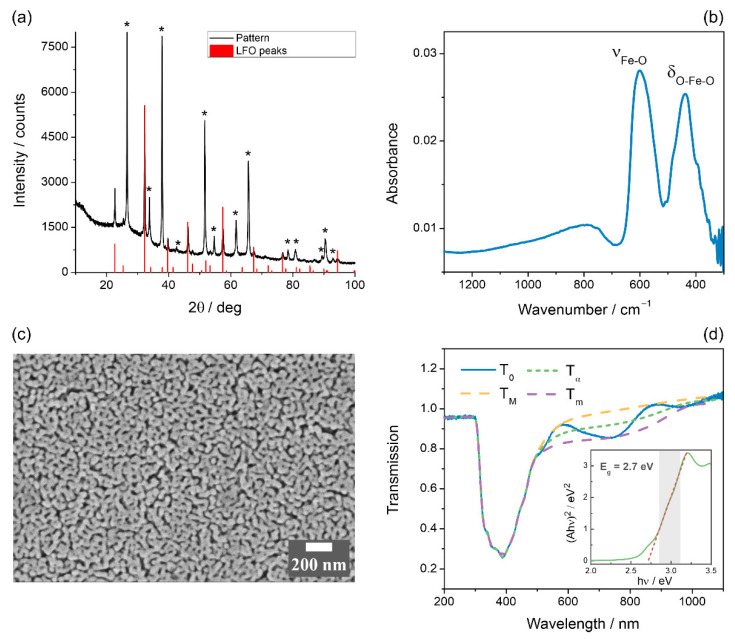
(**a**) XRD pattern of the LFO film on FTO. FTO peaks are marked with asterisks (*). LFO peaks are from ref. [46]. (**b**) ATR-FTIR spectrum of the LFO film. (**c**) SEM image of the LFO film. (**d**) Optical absorption spectrum of the LFO photoelectrode. The insert shows Tauc plot constructed to determine the band gap energy (E_g_).

Transparent LFO films were synthesized on FTO supports via spin coating of metal oxide precursors combined with Triton X-100 surfactant, which creates pores in the film upon decomposing [25]. The XRD pattern of the LFO film is given in Figure 1a. Except for the peaks from the substrate (FTO, marked with asterisks), the only phase present is the orthorhombic modification of LaFeO_3_ [46]. Characteristic peaks at 600 and 440 cm^−1^, corresponding to Fe–O stretching and O–Fe–O bending vibrations, were observed in the ATR-FTIR spectra (Figure 1b) of the prepared LFO films, which additionally proves the formation of the perovskite structure [47]. The synthesized LFO films demonstrate a porous morphology due to the polymer template (Figure 1c). The film is composed of grains with dimensions within the range of 50–150 nm, while the film thickness amounts to ca. 600 nm (as determined from an SEM image of the specimen cross-section, Figure S1). For LFO and similar materials, nanostructuring is considered essential to increase the active surface area and to reduce the diffusion length of charge carriers, which minimizes bulk recombination losses [23,48]. Figure S2 shows the XPS survey spectrum taken from the LFO film. The elemental composition given in Table S1 agrees well with the expected stoichiometry for LFO. No Sn3d line from tin in FTO supports was detected in the XPS spectra, which confirms the integrity of the film. The spectra in the O1s region reveal two well-separated peaks (Figure S3a). The peak (a) centered at 529.2 eV can be attributed to the lattice oxygen in the perovskite crystal structure [49,50]. The wide peak (b) at higher binding energy (531.3 eV) is a well-known feature of various perovskite XPS spectra, indicating the presence of a weakly bound oxygen, although its exact assignment to a certain oxygen-containing species is controversial [50,51].

The optical properties of the film were characterized by UV-vis spectroscopic measurements (Figure 1d shows the absorption spectrum of LFO). Optical absorption spectra of the prepared LaFeO_3_ photoelectrodes show an intense broad band with a maximum near 380 nm and a long low-absorbance tail at *λ* > 500 nm. It is worth noting that measured spectra are sufficiently affected by the fringes due to complex interference patterns in two thin films (FTO and LFO), subsequently covering the glass substrate. In order to avoid uncertainties (which may arise due to the measured absorption spectrum being distorted by interference fringes) in the optical band gap determination, the smooth fringe-devoid transmission curve *T_⍺_* was calculated and then used in the Tauc plotting. *T*_0_ in Figure 1d is the experimentally measured transmission curve. *T_M_* and *T_m_* are the envelopes, and splines are constructed as follows: in the region of weak or medium absorption (*λ* > 480 nm)—following the maxima (*T_M_*) or minima (*T_m_*) of the observed interference fringes; in the region of strong absorption (*λ* < 480 nm)—following the *T*_0_ curve. *T_⍺_* is the fringe-devoid transmission curve, calculated as *T_⍺_* = (*T_M_*·*T_m_*)^1/2^. The Tauc plot in the insert of Figure 1d is based on the *T_⍺_* curve, converted into absorbance units and then baseline-corrected (linear baseline). The band gap energy E_g_ was calculated to be 2.7 eV, which is close to the earlier estimates [26,31,34].

**Figure 2:**
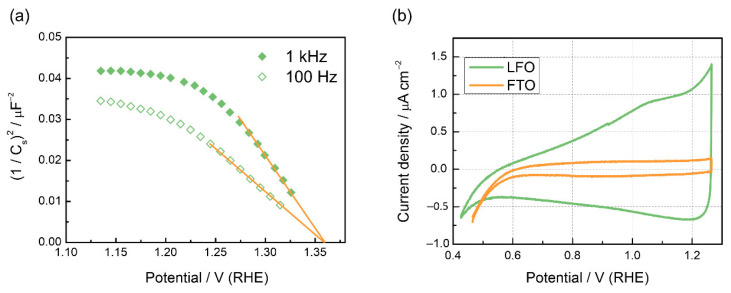
(**a**) Mott–Schottky plot for the LFO film in 0.1 M NaOH at 1000 Hz. (**b**) Dark voltammograms of the LFO film and bare FTO in 0.1 M NaOH at 10 mV s^−1^.

Mott–Schottky plots of the LFO film are displayed in Figure 2a. The negative slope confirms the *p*-type nature of the LaFeO_3_ material. The slope shows frequency dependence, which is typical for nanostructured films. From the linear segment of the plots, a flat band potential of 1.36 V was determined, which agrees well with the previous results for the non-doped LFO [23,31,34]. A cyclic voltammogram of a LaFeO_3_ film registered under dark conditions in 0.1 M NaOH is shown in Figure 2b, and the voltammogram of bare FTO support is given for comparison. LFO voltammograms show an increase in the capacitive current in the potential range 0.5–1.2 V, and the onset of oxygen evolution is observed at potentials higher than 1.3 V. A broad peak is observed at 1.0–1.05 V, which can be attributed to sub-bandgap surface states, which are linked to intrinsic defects such as cation vacancies [31]. One may also notice cathodic currents appearing at potentials more negative than 0.6 V vs. RHE for the FTO electrode, which refer to the irreversible reduction of tin oxide in alkaline media [52]. For the LFO film, no pronounced asymmetry in the cathodic and anodic charges is observed until ca. 0.4 V vs. RHE, although the reduction of the perovskite phase in alkaline media should occur at lower potentials. The limited cathodic stability of perovskite structures under alkaline conditions was previously revealed for Mn-based perovskites at potentials lower than 0.4 V vs. RHE [53].

### 3.2. Photoelectrochemical Properties

**Figure 3:**
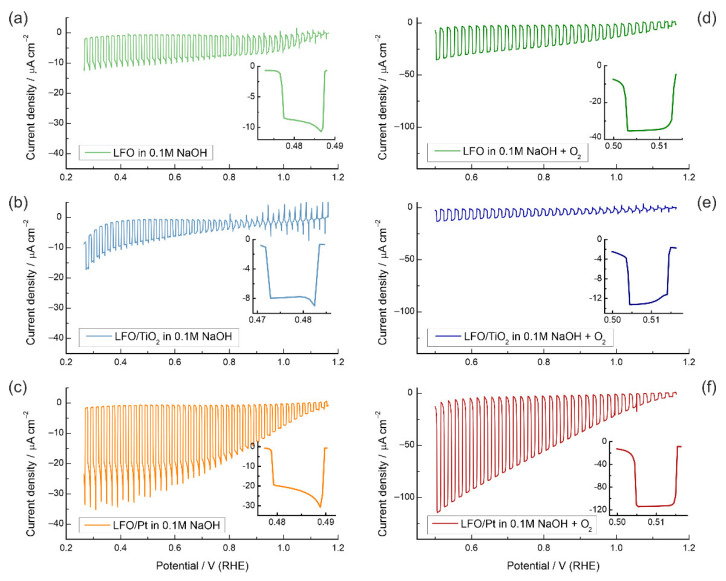
Linear sweep voltammograms at 5 mV s^−1^ under a square-wave 460 nm light perturbation for pristine LFO, LFO/TiO_2_(1:100) and LFO/Pt samples in deaerated 0.1 M NaOH (**a**–**c**) and O_2_-saturated 0.1 M NaOH (**d**–**f**). The inserts show the enlarged portions of the voltammograms in the 0.48–0.51 V range to illustrate the difference in current decays.

Figure 3a shows linear sweep voltammograms of an LFO electrode in a deaerated 0.1 M NaOH solution under chopped illumination (λ = 460 nm). The photocurrent at 0.5 V vs. RHE does not exceed ca. 10 µA cm^−2^, which reflects the low PEC activity of the pristine LFO film. Typical current decays after instantaneous rises in the illumination periods are indicative of rate-controlling recombination processes [54] (the inserts in Figure 3 show the enlarged portions of the voltammograms during a 2 s illumination period to illustrate the current decays). Apparently, these losses become more pronounced with decreasing potential, which shows that charge separation due to the increase in the overpotential does not suppress recombination, as would be expected for classical semiconducting electrodes.

To check the effect of surface passivation and suppression of surface recombination on the PEC activity enhancement for LFO, TiO_2_ layers were deposited on the surface of LFO from solutions with 1:8 (sample LFO/TiO_2_(1:8)) and 1:100 (sample LFO/TiO_2_(1:100)) titanium isopropoxide/acetylacetone molar ratios. An SEM image of the LFO/TiO_2_(1:100) electrode shows practically unaltered morphology of the film, without the presence of large TiO_2_ particles, which points to the uniformity of the passivating film (Figure S4). Figure S3b shows the high-resolution XPS spectrum of the LFO/TiO_2_(1:100) film, which confirms the coverage of the film surface with titanium oxide. The Ti2p is a doublet, with Ti2p_1/2_ at 464.0 eV and the Ti2p_3/2_ peak at 458.2 eV. These peak positions agree well with the literature data on TiO_2_ [55,56].

**Figure 4:**
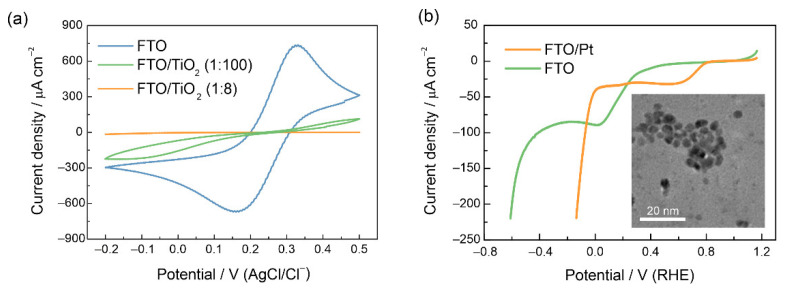
(**a**) Cyclic voltammograms at 25 mV s^−1^ of pristine FTO, FTO/TiO_2_(1:8), FTO/TiO_2_(1:100) electrodes in solution of 10 mM Fe(CN)_6_^3−^ in 0.5 M Na_2_SO_4_. (**b**) Linear sweep voltammograms of FTO electrode with and without Pt nanoparticles at 25 mV s^−1^. The insert shows TEM image of Pt nanoparticles.

The effect of TiO_2_ layers on blocking the electron transfer (ET) across the electrode/solution interface under dark conditions was examined by recording cyclic voltammograms of an FTO electrode with TiO_2_(1:8) and TiO_2_(1:100) coatings in a solution containing 0.5 M Na_2_SO_4_ and 10 mM Fe(CN)_6_^3−^ as a reversible redox probe (Figure 4a). One may notice that the TiO_2_(1:8) layer provides complete blocking of the ET rate to FTO, while TiO_2_(1:100) coating strongly reduces the ET rate but does not stop the tunneling completely.

Figure 3b shows linear sweep voltammograms of the LFO/TiO_2_(1:100) electrode under chopped illumination. Notably, the current at 0.5 V vs. RHE is only moderately diminished, which proves that the TiO_2_(1:100) coating does not suppress the HER. To prove that the TiO_2_ layer does not decrease the transmittance and the number of photogenerated charge carriers, we compared the voltammograms under front-side and back-side illumination, and the difference in current responses was found to be minor (Figure S5). The onset of photocathodic current is close for the pristine and TiO_2_-modified films (ca. 1.2 V), yet for the LFO electrode with the TiO_2_ layer, the photocurrent transients reveal spikes upon both illumination and light interruption until ca. 0.8 V. This is a signature of trapping of the photogenerated electrons at the surface, which are then consumed by fast recombination [57]. Figure S6 shows that the blocking TiO_2_(1:8) coating results in an even more pronounced surface trapping in the 0.8–1.2 V potential range and a 5-fold decrease in the photocurrent. Based on these experiments, we conclude that protective TiO_2_ layers do not enhance the PEC activity of LFO, and passivation of the recombination centers at the LFO surface should be regarded as an inefficient photocathode modification strategy. Since the TiO_2_ coating also suppresses the ET to the exposed FTO support, where the backward reaction may occur, we can conclude that the PEC activity of pristine LFO is not significantly affected by the possible backward hydrogen oxidation reaction (as was found to be the case for the BiVO_4_ photoanode [58]).

Next, we explored the effect of a Pt co-catalyst on the PEC activity of LFO electrodes. The LFO surface was modified with citrate-stabilized 5 nm-sized Pt nanoparticles, which were synthesized via a typical borohydride reduction route. XPS spectrum confirmed the presence of Pt nanoparticles on the surface of the LFO/Pt electrode (Figure S3c). The Pt4f region features a doublet with Pt4f_5/2_ at 74.5 eV and Pt4f_7/2_ at 71.2 eV, indicating that platinum particles are in the metallic state [59,60]. In addition, a prominent tail towards higher binding energies in the spectrum is observed. The shifting of the peaks to higher binding energies was observed previously for platinum nanoparticles of various sizes and is connected with the partial oxidation of the catalyst surface [61]. The deconvolution of the Pt4f spectrum has been performed to estimate shares of Pt^2+^ and Pt^4+^. The result of the Pt4f spectrum fitting with Pt4f_7/2_/Pt4f_5/2_ doublets is presented in Figure S7. The Pt 4f_7/2_-4f_5/2_ spin orbit splitting is 3.33 eV. The doublet with Pt4f_7/2_ located at 71.2eV is attributed to metallic platinum nanoparticles Pt(0). Two additional doublets with Pt4f_7/2_ binding energy located at 72.8 eV and 74.6 eV correspond to Pt^2+^ and Pt^4+^ states, respectively [62,63].

When Pt nanoparticles were deposited onto bare FTO supports, the catalytic effect in the dark manifested itself in a shift of the HER onset potential by ca. 0.5 V (Figure 4b). Notably, for the Pt-modified FTO electrode, the FTO reduction wave also shifts from ca. 0.2 V to 0.7 V, which points to the catalytic effect of Pt on the FTO reduction. The modification of the LFO surface with Pt nanoparticles results in a very noticeable increase in the photocurrent (ca. 30 µA cm^−2^), which is a threefold improvement over the unmodified LFO (Figure 3c). As one can see, the photocurrent decays are also more prominent, showing that the HER kinetics facilitated by Pt are still too slow to suppress surface recombination completely, and the photocurrent decreases rapidly from its instantaneous values. EIS data in Figure S8a confirm the observed trend*—*the highest resistance is observed for a TiO_2_-modified electrode, while the LFO/Pt sample shows the lowest resistance, which correlates with the currents in the voltammograms in Figure 3a–c.

**Figure 5:**
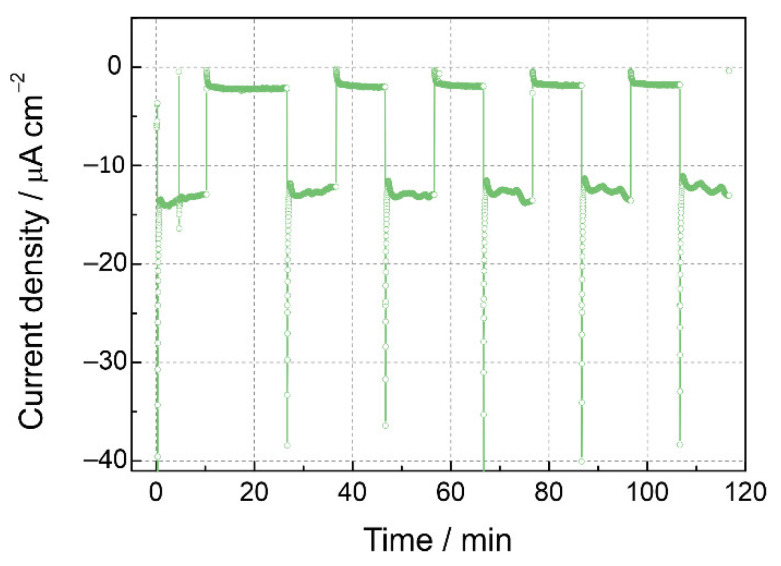
Stability test of LFO/Pt electrode at 0.4 V vs. RHE under chopped illumination in deaerated 0.1 M NaOH solution.

For the most active LFO/Pt sample, a stability test was carried out (Figure 5). Despite the initial current decay, no further diminution of the photocurrent density or growth of the dark currents were observed in the course of 2 h measurements under chopped illumination conditions. This can be regarded as the confirmation of the stability of the LFO phase under the reaction conditions and the assignment of the observed photocurrent to the hydrogen evolution [26,64] (and possibly reduction of traces of oxygen) rather than to the LFO photocorrosion current. 

### 3.3. Recombination and Charge Transfer Kinetics

**Figure 6:**
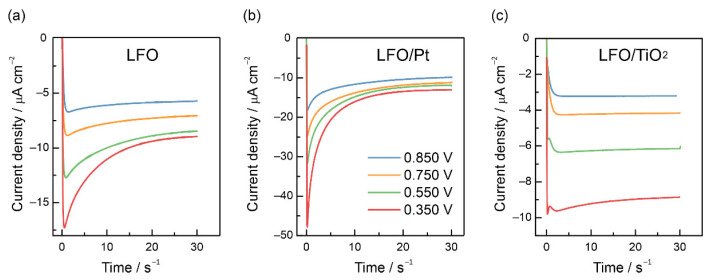
Potentiostatic photocurrent transients registered during the illumination periods for the LFO (**a**), LFO/Pt (**b**) and LFO/TiO_2_(1:100) (**c**) electrodes at potentials of 0.850, 0.750, 0.550, 0.350 V vs. RHE in deaerated 0.1 M NaOH solution.

Potentiostatic photocurrent transients were recorded to quantify the recombination losses. Figure 6a shows characteristic decaying transients registered at different potentials for a pristine LFO electrode. The decay of photocurrent with time reflects the build-up of photogenerated electrons at or near the interface, which causes a counter-flow of holes and provokes recombination. A steady state is achieved when the rate at which the electrons reach the interface is balanced by the rate at which they are consumed by charge transfer and recombination. For LFO/Pt electrodes, the steady state photocurrents increase, yet the current decays also become higher, which may suggest an increase in both the rate of charge transfer and recombination (Figure 6b). For the LFO/TiO_2_(1:100) electrode (Figure 6c), the photocurrent transients are much closer to simple rectangular “on-off” responses, which can be regarded as a consequence of low charge separation and low PEC activity. The current decays appear only at potentials more negative than 0.5 V.

Analysis of the photocurrent transients can be used to extract the values of recombination rate constant (*k*_rec_) and charge transfer rate constant (*k*_ct_) [54]:

(1) $$-\ln\left(\frac{i\left(t\right)-i_{\mathrm{ss}}}{i^{0}-i_{\mathrm{ss}}}\right)=\left(k_{\mathrm{tr}}+k_{\mathrm{rec}}\right)*t;\;$$

(2) $$\frac{i_{\mathrm{ss}}}{i^{0}}=\frac{k_{\mathrm{tr}}}{k_{\mathrm{tr}}+k_{\mathrm{rec}}}$$

where $i\left(t\right)$ is the time-dependent photocurrent density, *t* is the time, $i^{0}$ is the instantaneous photocurrent density, and $i_{\mathrm{ss}}$ is the steady-state photocurrent density. In this case, the decrease in the photocurrent decay and the increase in the steady-state photocurrent would signify reduced recombination. Notably, the procedure for recombination rate constant determination from photocurrent transients is only valid at short times since, at longer times, the deviations related to the build-up of minority charge carriers at the surface appear, which might alter band bending. Figure S9 shows examples of the linearization of the $\ln\left(\frac{i\left(t\right)-i_{\mathrm{ss}}}{i^{0}-i_{\mathrm{ss}}}\right)$ vs. time plots, which were used to derive information on the values of *k*_tr_ and *k*_rec_.

**Figure 7:**
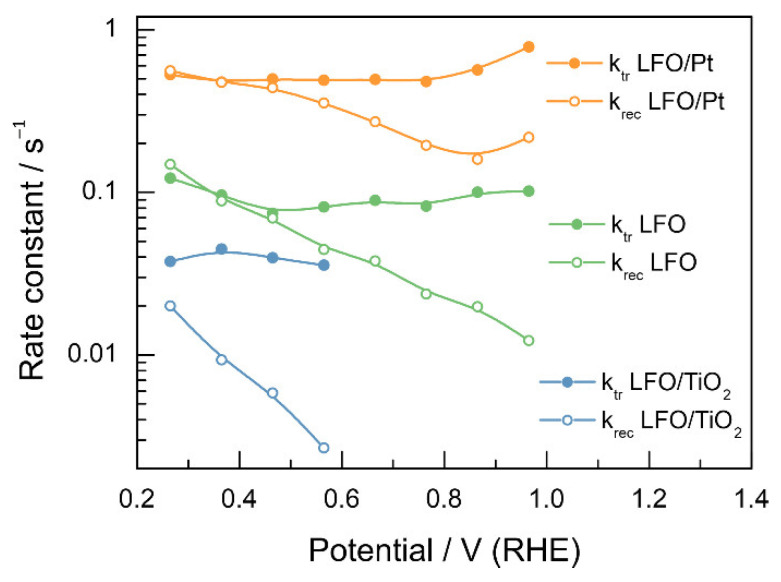
Potential dependence of the charge transfer rate constant and recombination rate constant, as determined from transient photocurrent measurements.

Figure 7 shows the recombination and charge transfer rate constants (Table S2 collects the values of rate constants). On average, the charge transfer rate constant for LFO/Pt is ca. 5 times higher than that for LFO without the Pt nanoparticles, which shows that the co-catalyst is essential to enhance the charge transfer rate. The recombination rate constants are expected to decrease with the increase in the overpotential due to better charge separation associated with the changes in band bending [40]. However, for the LFO and LFO/Pt electrodes, the opposite trend is observed*—*the recombination rate constants show exponential growth with a change in potential from 1.0 V to 0.3 V vs. RHE. Effectively, this means that the charge transfer efficiency decreases with the increase in the overpotential. Recombination rate constants are ca. 7 times higher for LFO/Pt, which shows that the co-catalyst does not reduce recombination, although it enhances the charge transfer. For LFO/TiO_2_, the charge transfer rate constant decreases by a factor of 2 compared to pristine LFO, while the recombination rate constants drop by an order of magnitude, which is the expected behavior for a passivating coating. Notably, at potentials less positive than 0.5 V, both k_rec_ and k_tr_ for pristine LFO and LFO/Pt increase with the decrease in potential, which might be related to the changes in the potential drop across the Helmholtz layer (compact part of the double layer [65]).

In the next step, we explored the kinetics of photoelectrochemical processes on modified LFO electrodes for a reactant with more facile charge transfer kinetics. For LaFeO_3_-based photocathodes, dissolved oxygen is often used as an electron scavenger, with the ET to O_2_ proceeding at a higher rate compared to ET to water molecules in alkaline media [23,31,34]. For pristine LFO, the current for O_2_ reduction is ca. 3.5 times higher than the current for water reduction (Figure 3d), while for TiO_2_-covered LFO, the increase only amounts to 1.5 times (Figure 3e), which implies a reduction of the ET rate due to passivation. For the LFO/Pt electrodes, the currents are enhanced by a factor of four (Figure 3f). No typical signs of surface recombination can be observed in the voltammograms for O_2_ reduction; the currents are steadily growing during the illumination periods. These results show that compared to the case of water reduction, the surface recombination is minor. Figure S10 shows characteristic photocurrent transients, registered in O_2_-saturated 0.1 M NaOH solution for LFO/Pt electrodes, with stable photocurrents without the current spikes and subsequent decays (small current decays appear only at potentials less positive than 0.4 V vs. RHE, which is a significant improvement over the trend observed for water reduction). EIS data in Figure S8b reveal characteristic semicircles in the Nyquist plots for LFO, LFO/TiO_2_ and LFO/Pt, with the smallest diameter being observed for the LFO/Pt sample, which confirms much faster charge transfer kinetics for the LFO photoelectrode with a Pt co-catalyst.

**Figure 8:**
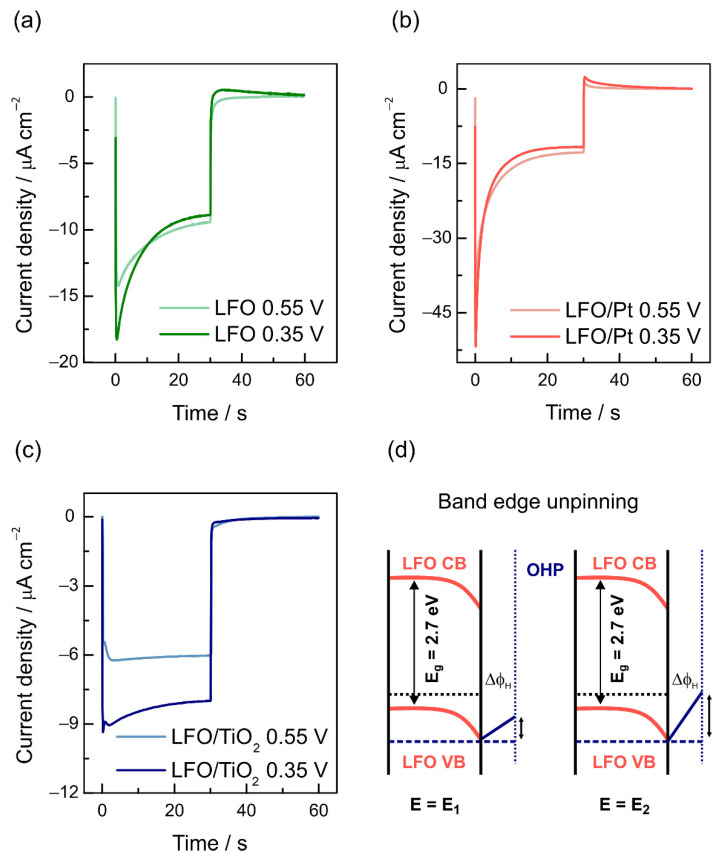
Potentiostatic current transients for LFO (**a**), LFO/Pt (**b**) and LFO/TiO_2_ (**c**) electrodes at potentials of 0.550 and 0.350 V vs. RHE under chopped illumination. (**d**) Schematic illustration of band edge unpinning in LaFeO_3_ electrode.

To explore the effect of surface coating on the hydrogen evolution kinetics further, we analyzed the dark responses of the LFO films registered after switching off the illumination (Figure 8). Notably, for the LFO and LFO/Pt electrodes, the differences in current responses at 0.35 and 0.55 V are minor (Figure 8a,b) yet become very pronounced for the LFO/TiO_2_ sample (Figure 8c). These differences correlate with current vs. time responses in Figure 3a–c, where the currents for the LFO and LFO/Pt electrodes are very close at 0.35 and 0.55 V, while for the LFO/TiO_2_ electrodes, the current increases significantly when changing the potential from 0.55 V to 0.35 V. This shift correlates with the negative shift of the hydrogen evolution onset potential for the passivated LFO/TiO_2_ surface.

It is expected that the decrease in cathodic photocurrent due to recombination up to the point where the light is switched off should be equal in magnitude to the instantaneous anodic spike, which appears due to the flow of holes to recombine with the electrons accumulated in the surface region [66]. For the LFO and LFO/Pt electrodes, the cathodic photocurrent decays after the initial spike, but there is only a small overshoot. For LFO/TiO_2_, the overshoot is practically invisible. The discrepancy between the magnitudes of cathodic current decay and anodic current overshoot can be explained by the light-induced band edge unpinning due to the build-up of electrons at the interface, which modifies the potential distribution across the LFO/electrolyte interface [54,57,66]. The potential drop across the Helmholtz layer (Δϕ_H_, Figure 8d) increases at the expense of decreasing the potential drop across the space charge region, and the decreased band bending causes decay of the photocurrent. For LaFeO_3,_ the observed effect could also correspond to the reduction of surface Fe(III) states to Fe(II) states, and recombination would then be attributed to the reoxidation of Fe(II) by holes. However, since the long-term tests imply the stability of the steady-state photocurrent, we assume that such a reduction only involves the surface atoms.

## 4. Discussion

The observed trends allow us to speculate on the strategies to overcome the limitations in the use of LFO electrodes for PEC applications that hinder the advancement of this material toward commercial PEC devices. We found that the activity of a nanoporous LFO film with a Pt co-catalyst exceeds that reported for the doped LaFeO_3_ films, both in HER and ORR (Table S3 compares our results with the literature data). Ca. 50–75 µA cm^−2^ at 0.5 V vs. RHE were achieved in O_2_-saturated alkaline solutions for Zn-, Mg-, Ca-, Sr- or Ba-doped LFO films [31,34], while in our study, the photocurrents for the non-doped LFO with a Pt co-catalyst at 0.5 V exceed 100 µA cm^−2^. In deaerated alkaline solutions, the stationary photocurrents for the doped LFO films still do not exceed several µA cm^−2^, while for the LFO/Pt film, tens of µA cm^−2^ were obtained in this study. Much higher photocurrent densities with oxygen as an electron scavenger (up to 200 µA cm^−2^) were reported only for the LaFeO_3_ film prepared by electrodeposition, yet these were thick and non-transparent deposits with a surface area much larger than that characteristic for transparent LFO film synthesized in this work [23,33]. Moreover, the photocurrents in deoxygenated alkaline solutions for such electrodeposited films did not exceed 10 µA cm^−2^, which is comparable with the performance of the obtained LFO film. It can be expected that the PEC activity of the electrodeposited LFO will also improve significantly with the addition of a co-catalyst.

The results of previous studies [34] indicate that doping of the LFO structure does not lead to a noticeable increase in the charge transfer rate. The effect of doping is primarily associated with an increase in majority carrier concentration resulting from a dopant-induced increase in the Fe^4+^ density caused by charge compensation upon substitution of Fe^3+^ by divalent ions and, possibly, with the increase in the hole mobility [34]. We suggest that further development of LaFeO_3_-based photocathodes should combine co-catalyst deposition with the doping strategy to reduce bulk recombination by facilitating electron-hole separation.

## 5. Conclusions

The photoelectrochemical properties of a nanostructured LaFeO_3_ film were demonstrated to depend greatly on the presence of a passivating coating or a co-catalyst. We found that although LFO films show quite poor activity in HER, the photocurrents can be increased by a factor of three, reaching 30 µA cm^−2^ if Pt nanoparticles are deposited onto the film surface. A protective TiO_2_ layer does not provide any increase in the PEC activity due to surface passivation. As the LFO electrodes exhibit high stability under illumination in aqueous alkaline media, the additional surface passivation seems to be unnecessary, while the addition of a co-catalyst was found to be essential to enhance both the HER and ORR kinetics. ORR currents increased by a factor of 4 when a Pt co-catalyst was deposited onto the film, which demonstrated the importance of increasing the rate of interfacial charge transfer for LFO photocathodes. For slower HER, the build-up of electrons at the interfaces translates into band edge unpinning, which reduces the photocurrent. Our findings suggest that the nanostructured LFO photoelectrode does not require surface protection for the development of this material for PEC water-splitting applications, while the utilization of a co-catalyst is essential for enhancing the PEC activity.

## Data Availability

The data presented in this study are available on request from the corresponding author.

## Supplementary Materials

The following supporting information can be downloaded at: https://www.mdpi.com/article/10.3390/nano12234327/s1, Figure S1: SEM image of FIB cross-section of LFO film. Figure S2: XPS survey spectra taken from LFO, LFO/Pt and LFO/TiO_2_ electrodes. Table S1: Surface element concentrations on the electrode surface calculated based on the XPS data. Figure S3: High-resolution XPS spectra: O1s spectra for LFO, LFO/Pt and LFO/TiO_2_ films, Ti2p spectrum for LFO/TiO_2_ film, and Pt4f spectrum for LFO/Pt film. Figure S4: SEM image of LFO/TiO_2_(1:100) electrode. Figure S5: Linear sweep voltammograms at 5 mV s−1 under a square-wave 460 nm light perturbation for pristine LFO/TiO_2_(1:100) in deaerated 0.1 M NaOH under front-side and back-side illumination. Figure S6: Linear sweep voltammograms at 5 mV s−1 under a square-wave 460 nm light perturbation for pristine LFO, LFO/TiO_2_(1:8) and LFO/TiO_2_(1:100) samples in deaerated 0.1 M NaOH. Figure S7. Pt4f spectrum fitting. Figure S8: Impedance spectra of LFO/TiO_2_(1:100), LFO and LFO/Pt electrodes registered under illumination in deaerated 0.1 M NaOH and in O_2_-saturated 0.1 M NaOH. Figure S9: Linearized $\ln\left(\frac{i\left(t\right)-i_{\mathrm{ss}}}{i^{0}-i_{\mathrm{ss}}}\right)$ vs. time plots for LFO, LFO/Pt and LFO/TiO_2_ at 0.55 V vs. RHE. Table S2: Charge transfer rate constants and recombination rate constants determined from transient photocurrent measurements for LFO, LFO/Pt and LFO/TiO_2_ electrodes. Figure S10: Potentiostatic photocurrent transients registered during the illumination periods for the LFO/Pt electrodes at potentials of 0.850, 0.750, 0.550, 0.350 V vs. RHE in O2-saturated 0.1 M NaOH solution. Table S3. Comparison of the photocurrent densities with the literature data. Reference [67] is cited in the supplementary materials.
