# Supplementary material for: Evaluation of the Efficiency of Photoelectrochemical Activity Enhancement for the Nanostructured LaFeO3 Photocathode by Surface Passivation and Co-Catalyst Deposition
Source: Nanomaterials (Basel). 2022 Dec 5;12(23):4327. doi: 10.3390/nano12234327 (PMC9741200; doi:10.3390/nano12234327)
Supplement: Supplementary file 1 [file nanomaterials-12-04327-s001.zip › nanomaterials-2058027-supplementary.pdf]

Supplementary materials

Evaluation of the efficiency of photoelectrochemical activity enhancement for the nanostructured $LaFeO_3$ photocathode by surface passivation and co-catalyst deposition

Victoria P. Chertkova,[1] Aleksandra N. Iskortseva,[1,2] Egor M. Pazhetnov,[2] Sergey V. Ryazantsev,[2,1] Natalia A. Arkharova,[3] Eduard E. Levin,[1,3] Victoria A. Nikitina[2,1]*

[1]Department of Chemistry, Lomonosov Moscow State University, Moscow 119991, Russian Federation

[2]Center for Energy Science and Technology, Skolkovo Institute of Science and Technology, Moscow 121205, Russian Federation

[3]FSRC "Crystallography and Photonics" RAS, 119333, Moscow, Russian Federation

*Corresponding author: v.nikitina@skoltech.ru

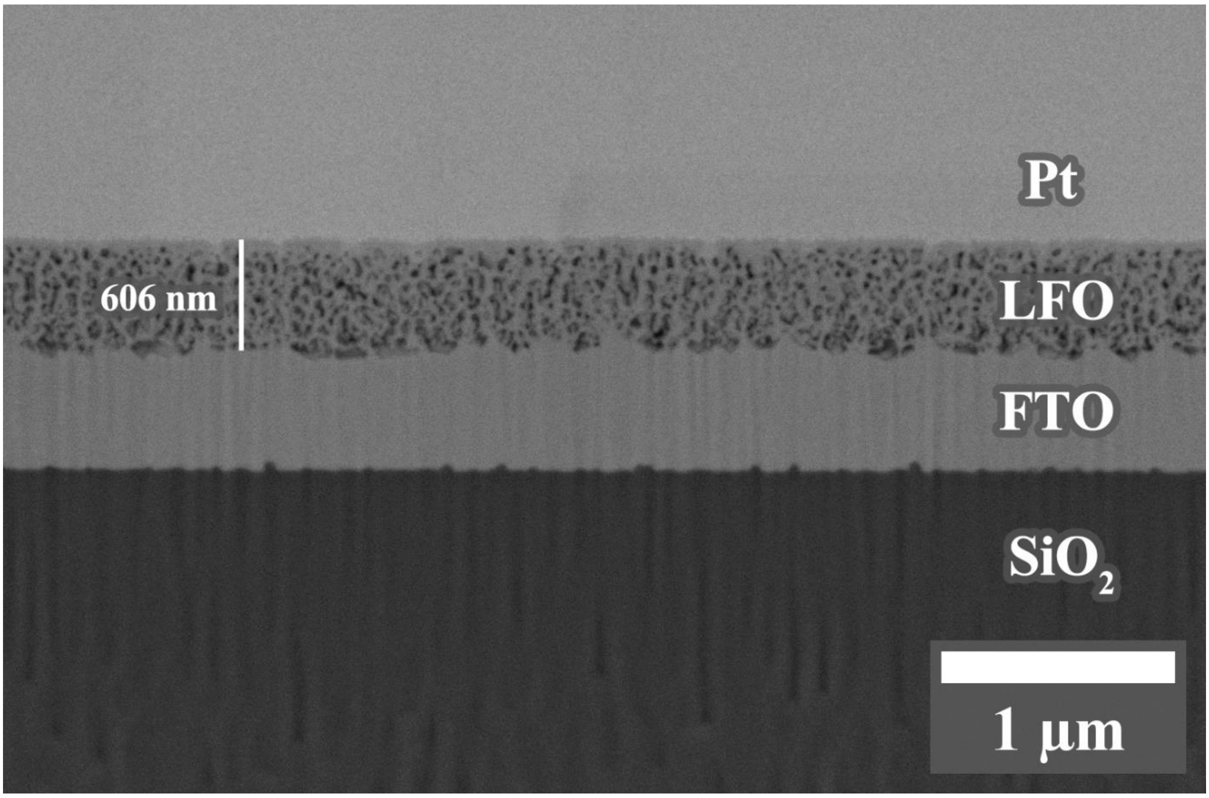

Figure S1. SEM image of FIB cross section of LFO film. Denoted thickness of the film is corrected for specimen tilt.

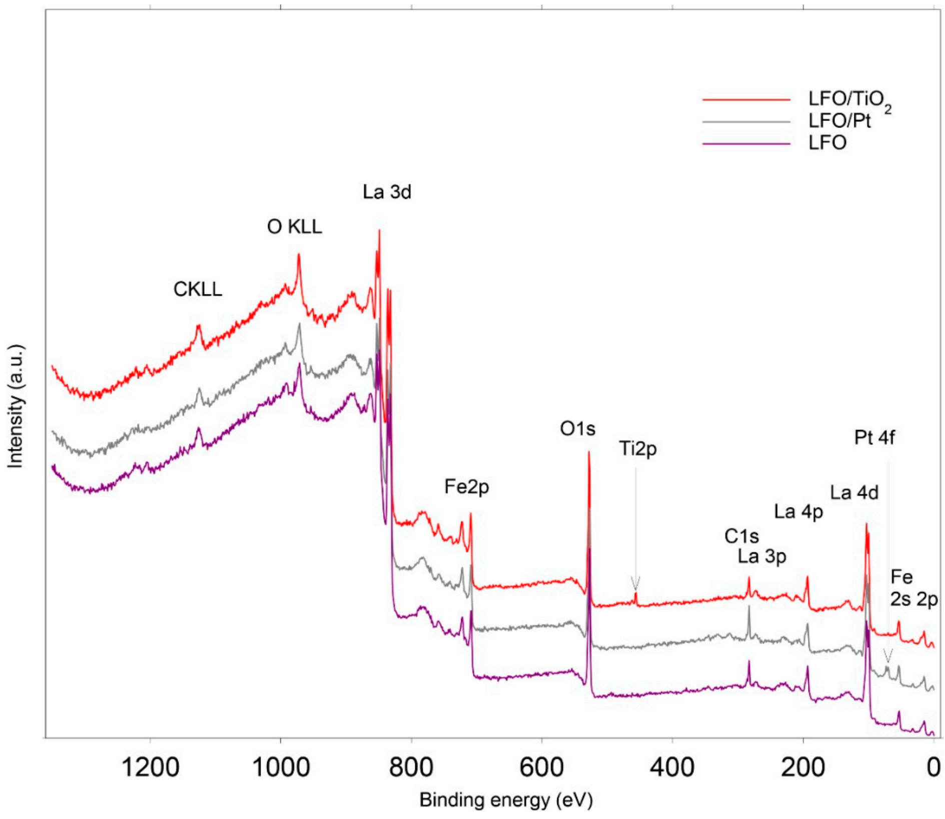

Figure S2. XPS survey spectra taken from LFO, LFO/Pt and LFO/TiO_2 electrodes. Spectra were shifted along the Y-axis for presentation.

Table S1. Surface element concentrations on the electrode surface calculated based on the XPS data.

Sample	C, at%	O, at%	La, at%	Fe, at%	Ti, at%	Pt, at%
LFO	22.3	54.9	11.4	11.4	ND	ND
LFO/Pt	28.3	49.8	10.3	10.7	ND	0.9
LFO/TiO_2	18.0	55.9	12.2	12.1	1.8	ND

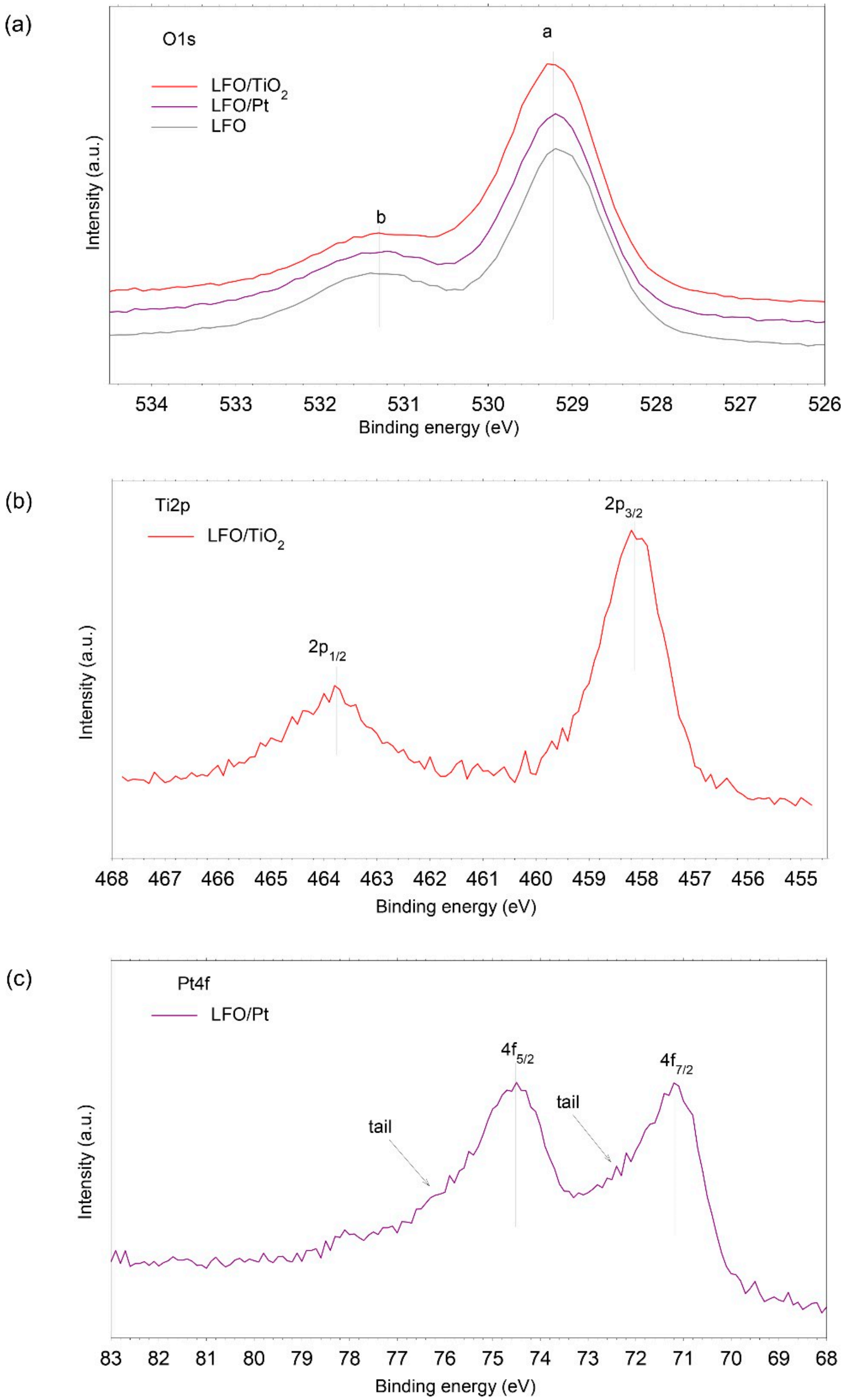

Figure S3. High resolution XPS spectra: O1s spectra for LFO, LFO/Pt and LFO/TiO_2 films (a), Ti2p spectrum for LFO/TiO_2 film (b), and Pt4f spectrum for LFO/Pt film (c).

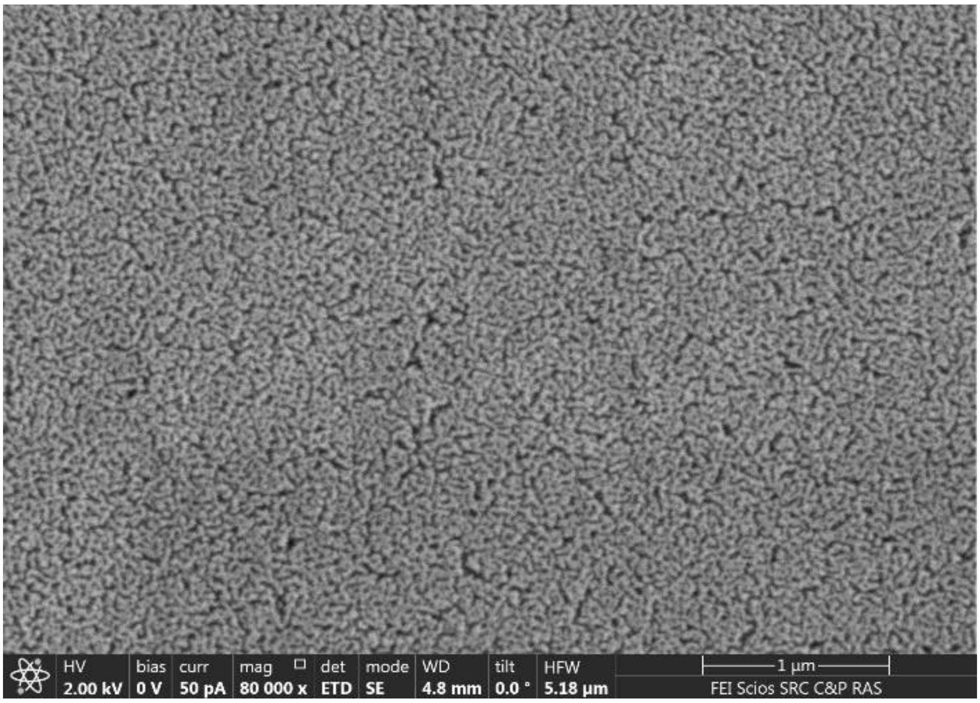

Figure S4. SEM image of LFO/TiO_2(1:100) electrode.

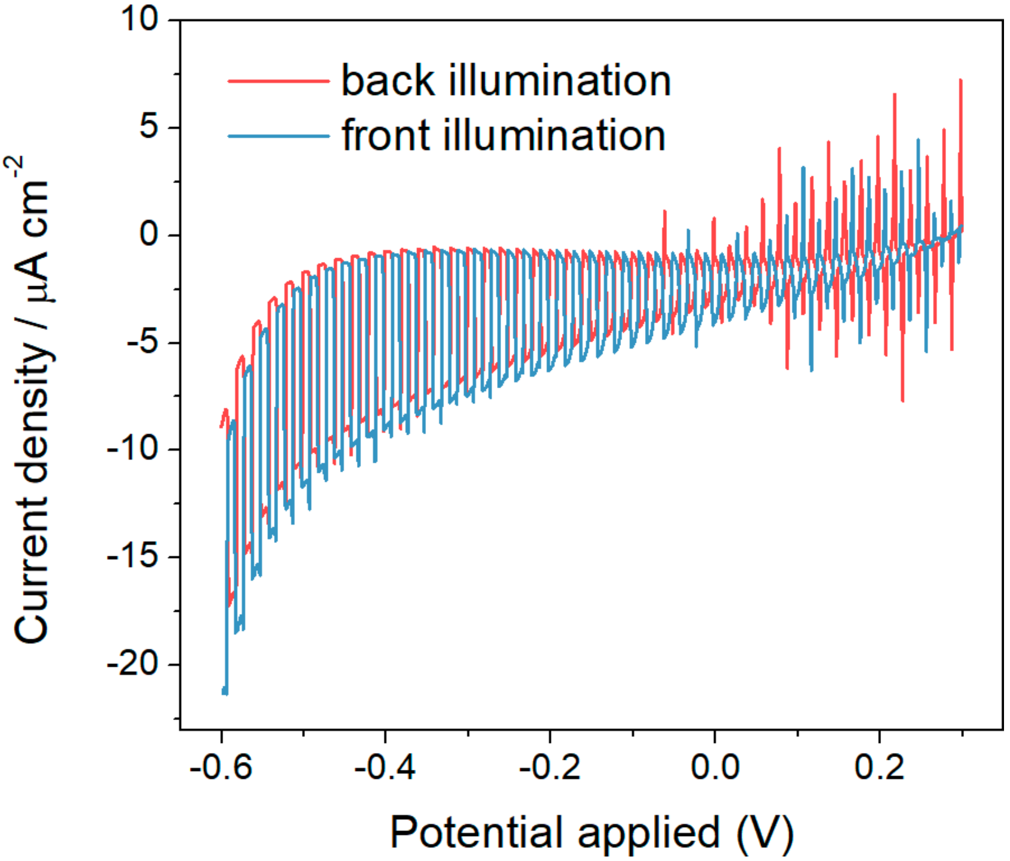

Figure S5. Linear sweep voltammograms at 5 mV s^{-1} under a square-wave 460 nm light perturbation for pristine LFO/TiO_2(1:100) in deaerated 0.1 M NaOH under front-side and back-side illumination.

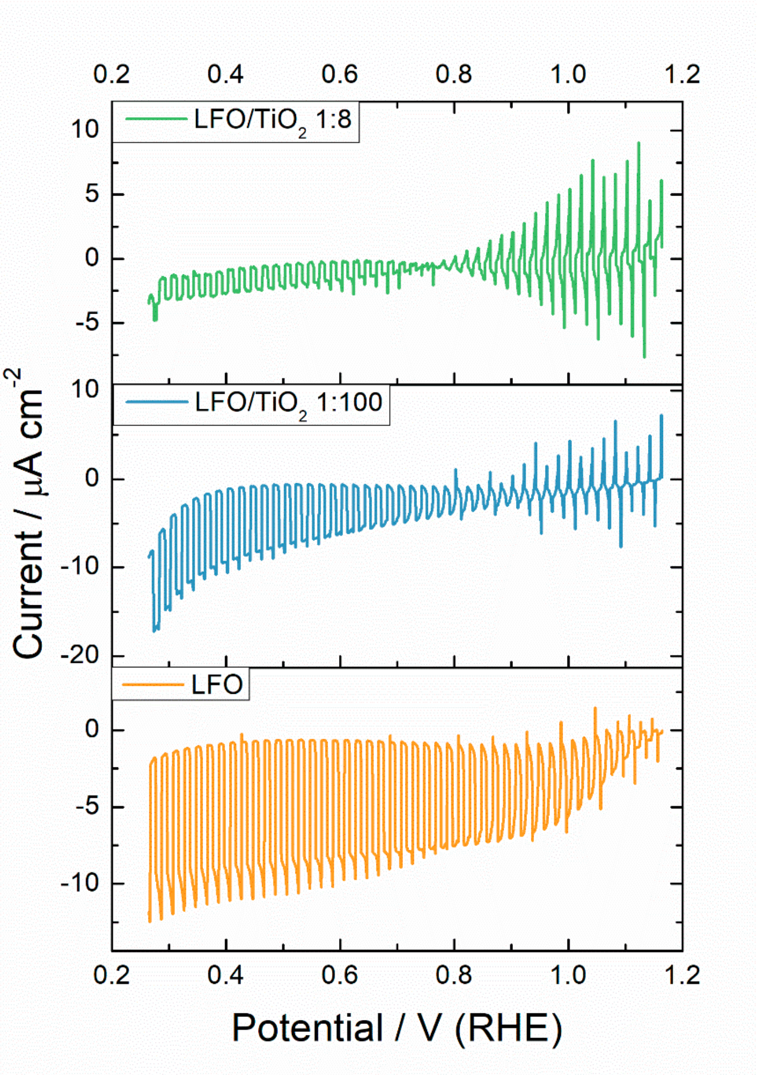

Figure S6. Linear sweep voltammograms at 5 mV s^{-1} under a square-wave 460 nm light perturbation for pristine LFO, LFO/TiO_2(1:8) and LFO/TiO_2(1:100) samples in deaerated 0.1 M NaOH.

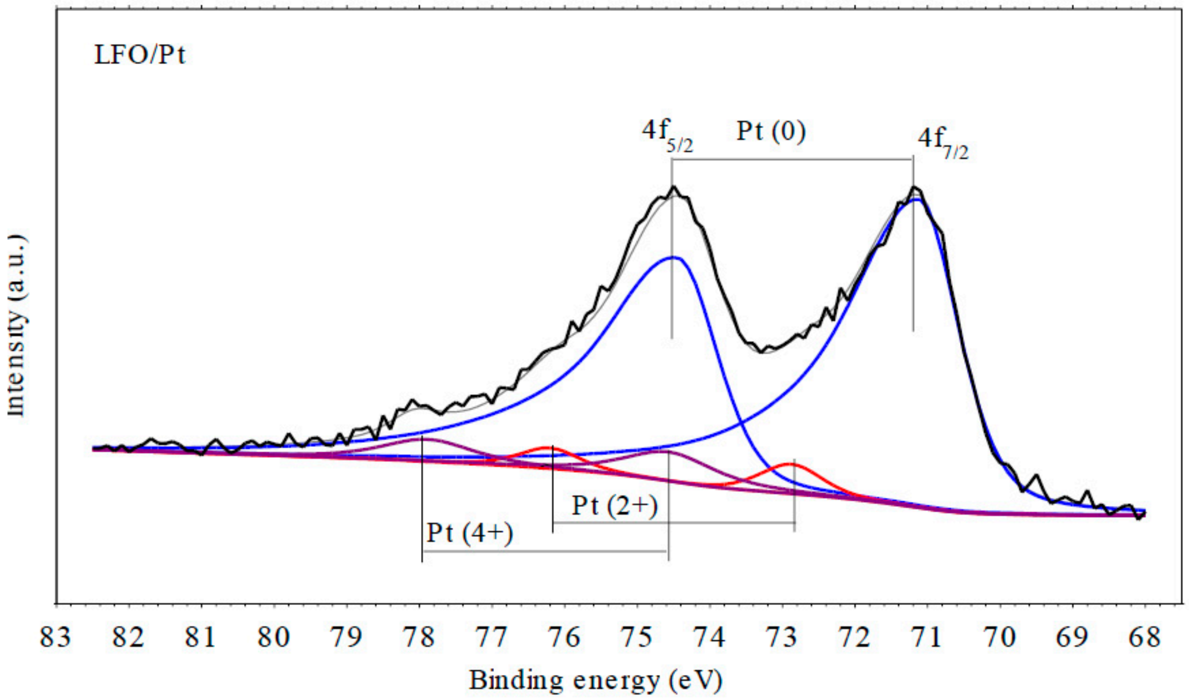

Figure S7. Pt4f spectrum fitting.

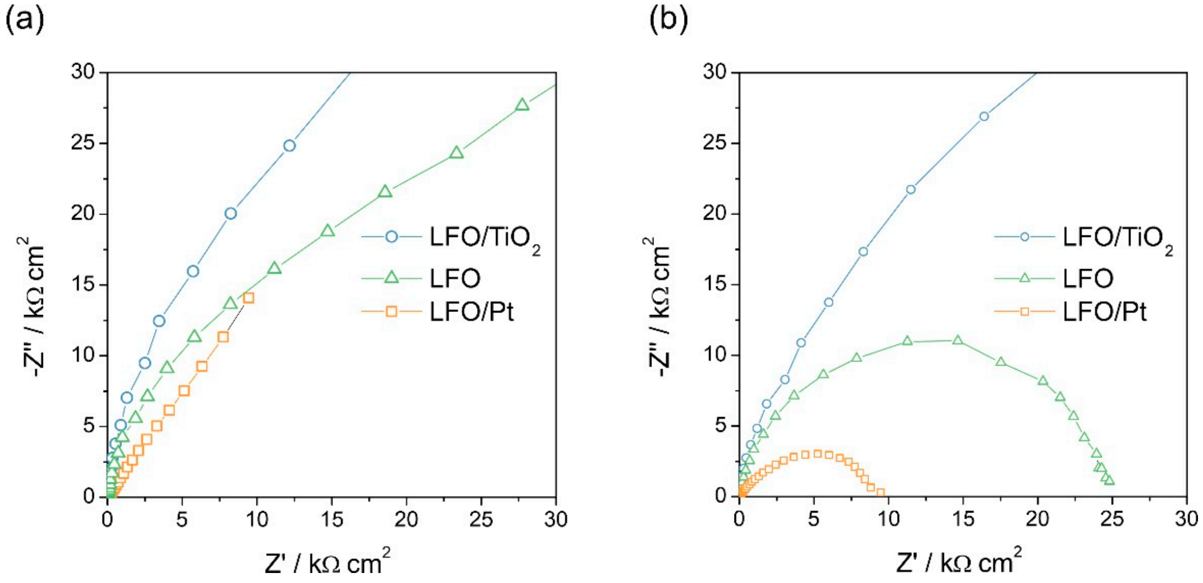

Figure S8. Impedance spectra of LFO/TiO_2(1:100), LFO and LFO/Pt electrodes registered under illumination in deaerated 0.1 M NaOH (a) and in O_2-saturated 0.1 M NaOH (b).

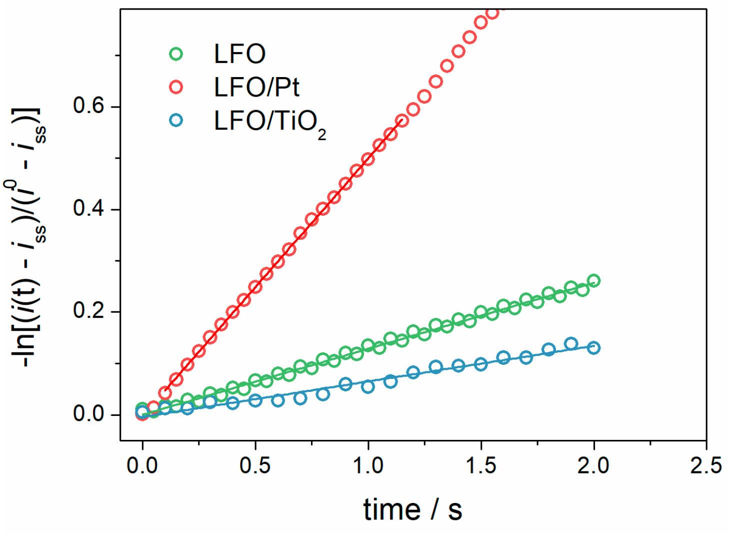

Figure S9. Linearized $\ln\left(\frac{i(t)-i_{ss}}{i^0-i_{ss}}\right)$ vs. time plots for LFO, LFO/Pt and LFO/TiO_2 at 0.55 V vs. RHE. Symbols are experimental data points, lines are linear approximations.

Table S2. Charge transfer rate constants and recombination rate constants determined from transient photocurrent measurements for LFO, LFO/Pt and LFO/TiO_2 electrodes.

	LFO		LFO / Pt		LFO / TiO_2	
E, V	k_{tr}, s^{-1}	k_{rec}, s^{-1}	k_{tr}, s^{-1}	k_{rec}, s^{-1}	k_{tr}, s^{-1}	k_{rec}, s^{-1}
0.95	0.102	0.012	0.783	0.218	-	-
0.85	0.100	0.020	0.565	0.160	-	-
0.75	0.082	0.024	0.480	0.194	-	-
0.65	0.089	0.038	0.494	0.271	-	-
0.55	0.081	0.044	0.489	0.354	0.036	0.003
0.45	0.074	0.069	0.498	0.440	0.039	0.006
0.35	0.096	0.089	0.476	0.473	0.045	0.009
0.25	0.122	0.149	0.529	0.558	0.038	0.020

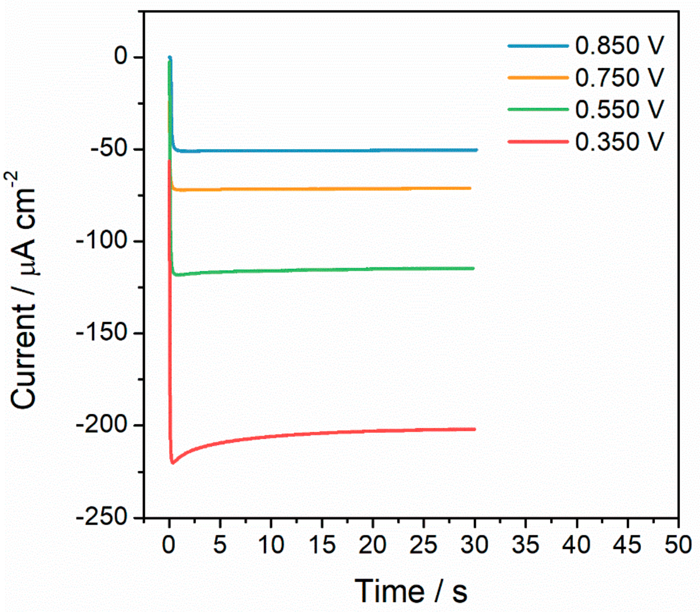

Figure S10. Potentiostatic photocurrent transients registered during the illumination periods for the LFO/Pt electrodes at potential 0.850, 0.750, 0.550, 0.350 V vs. RHE in O_2-saturated 0.1M NaOH solution.

Table S3. Comparison of the photocurrent densities with the literature data.

Sample composition	Solution	Photocurrent density at 0.5 V vs. RHE in deaerated solution	Photocurrent density at 0.5 V vs. RHE in O_2 saturated solution	Ref.
LFO	0.1 M NaOH	10 µA cm^2	35 µA cm^2	This work
LFO/Pt		30 µA cm^2	115 µA cm^2	
LFO	0.1 M Na_2SO_4 (pH 12)	0.1 µA cm^2	-	[1]
LFO/TiO_2/Pt		1.5 µA cm^2	-	
Mg-doped LFO	0.1 M Na_2SO_4 (pH 12)	ca. 1 µA cm^2	30 µA cm^2	[2]
Zn-doped LFO	0.1 M NaOH	-	60 µA cm^2	[3]
LFO		ca. 1 µA cm^2	10 µA cm^2	
LFO	0.1 M KOH	10 µA cm^2	25 µA cm^2	[4]
Li-doped LFO		30 µA cm^2	70 µA cm^2	
LFO (electrodeposited)	0.1 M NaOH	10 µA cm^2	200 µA cm^2	[5]

References

1. Sun, X., Tiwari, D., Fermin, D.J., Nanostructured $LaFeO_3$ Photocathodes with Onset Potentials for the Hydrogen Evolution Reaction Over 1.4 V vs. RHE, *J. Electrochem. Soc.*, **2019**, *166,* H764-H768.
2. Sun, X., Tiwari, D., Fermin, D.J., Promoting Active Electronic States in $LaFeO_3$ Thin-Films Photocathodes via Alkaline-Earth Metal Substitution, *ACS Appl. Mater. Interfaces*, **2020**, *12,* 31486-31495.
3. Diez-Garcia, M.I., Gomez, R., Metal Doping to Enhance the Photoelectrochemical Behavior of $LaFeO_3$ Photocathodes, *ChemSusChem*, **2017**, *10,* 2457-2463.
4. Li, Y., Wang, T., Gao, B., Fan, X., Gong, H., Xue, H., Zhang, S., Huang, X., He, J., Efficient photocathode performance of lithium ion doped $LaFeO_3$ nanorod arrays in hydrogen evolution, *New J. Chem.*, **2021**, *45,* 3463-3468.
5. Wheeler, G.P., Choi, K.-S., Photoelectrochemical Properties and Stability of Nanoporous p-Type $LaFeO_3$ Photoelectrodes Prepared by Electrodeposition, *ACS Energy Lett.*, **2017**, *2,* 2378-2382.
